# Intrinsic Toxin-Derived Peptides Destabilize and Inactivate *Clostridium difficile* TcdB

**DOI:** 10.1128/mBio.00503-17

**Published:** 2017-05-16

**Authors:** Jason L. Larabee, Sarah J. Bland, Jonathan J. Hunt, Jimmy D. Ballard

**Affiliations:** Department of Microbiology and Immunology, University of Oklahoma Health Sciences Center, Oklahoma City, Oklahoma, USA; University of Pittsburgh School of Medicine

**Keywords:** *Clostridium difficile*, TcdB, exotoxins, peptides

## Abstract

*Clostridium difficile* infection (CDI) is a major cause of hospital-associated, antibiotic-induced diarrhea, which is largely mediated by the production of two large multidomain clostridial toxins, TcdA and TcdB. Both toxins coordinate the action of specific domains to bind receptors, enter cells, and deliver a catalytic fragment into the cytosol. This results in GTPase inactivation, actin disassembly, and cytotoxicity. TcdB in particular has been shown to encode a region covering amino acids 1753 to 1851 that affects epitope exposure and cytotoxicity. Surprisingly, studies here show that several peptides derived from this region, which share the consensus sequence _1769_NVFKGNTISDK_1779_, protect cells from the action of TcdB. One peptide, PepB2, forms multiple interactions with the carboxy-terminal region of TcdB, destabilizes TcdB structure, and disrupts cell binding. We further show that these effects require PepB2 to form a higher-order polymeric complex, a process that requires the central GN amino acid pair. These data suggest that TcdB_1769–1779_ interacts with repeat sequences in the proximal carboxy-terminal domain of TcdB (i.e., the CROP domain) to alter the conformation of TcdB. Furthermore, these studies provide insights into TcdB structure and functions that can be exploited to inactivate this critical virulence factor and ameliorate the course of CDI.

## IMPORTANCE

*Clostridium difficile* is a leading cause of hospital-associated illness that is often associated with antibiotic treatment. To cause disease, *C. difficile* secretes toxins, including TcdB, which is a multidomain intracellular bacterial toxin that undergoes conformational changes during cellular intoxication. This study describes the development of peptide-based inhibitors that target a region of TcdB thought to be critical for structural integrity of the toxin. The results show that peptides derived from a structurally important region of TcdB can be used to destabilize the toxin and prevent cellular intoxication. Importantly, this work provides a novel means of toxin inhibition that could in the future develop into a *C. difficile* treatment.

## INTRODUCTION

TcdB is an intracellular bacterial toxin produced by the spore-forming, anaerobic, human pathogen *Clostridium difficile* (1). This 2,366-amino-acid (aa) protein binds to cell surface receptors (CSPG4, PVRL3, and FZD), undergoes endocytosis, and translocates into the cytosol, where it glucosylates small GTPases (2–5). Small-angle X-ray scattering, electron microscopy, and biochemical analyses indicate that TcdB is organized into 4 domains, with an amino-terminal glucosylation domain (aa 1 to 543), a cysteine-protease domain (aa 544 to 801), a cell entry/translocation domain (aa 802 to 1650), and a carboxyl-terminal cell-binding domain (aa 1651 to 2366) that includes combined repetitive oligopeptide repeats (CROP) from aa 1830 to 2366 (6–15). More recently, the crystal structure of TcdA, a protein that shares sequence and functional identity with TcdB, was solved and revealed a distinct structural organization associated with the biochemical activities of the protein (16). Specific cellular cues such as endosomal pH and cellular inositol hexakisphosphate (IP6) trigger conformational changes that lead to exposure of hydrophobic domains and autoprocessing of TcdB during distinct steps in cell entry. Premature activation of these functions renders the toxin inactive; for example, activation of autoprocessing prior to cell entry prevents TcdB cytotoxicity (17). Thus, TcdB is organized into functional domains that must become activated or exposed at precise steps during cell entry in order for the toxin to successfully complete intoxication. An unexplored vulnerability of TcdB may be the use of select molecules that cause premature conformational changes in the toxin and thereby prevent toxicity.

One promising destabilizing target in TcdB is the region proximal to the CROP domain that spans amino acids 1753 to 1852. This region of TcdB appears to be a critical structural linchpin that is involved in cell entry and structural integrity. For instance, an internal deletion mutant lacking amino acids 1756 to 1852 is nontoxic and unable to translocate into cells (14). Smaller internal deletions within this region cause TcdB to expose hydrophobic regions and become more sensitive to acid-pH-induced conformational changes (18). In previous studies, we examined the ability of this region to influence epitope exposure in distal portions of the toxin (19). During comparisons of the 1753-to-1851 region between variant forms of TcdB, we found that this portion of TcdB2 (produced by the 027 ribotype, toxinotype III) mediates intratoxin interactions and shields neutralizing epitopes that are otherwise exposed in TcdB1 (common to multiple ribotypes, toxinotype 0). This same region in TcdB1 is unable to form strong intramolecular contacts like those found in TcdB2. By comparing the variant amino acids in the 1753-to-1851 region of TcdB1 and TcdB2 as well as making appropriate amino acid swaps, two 8-amino-acid segments were identified in TcdB2 (1773 to 1780 and 1791 to 1798) that were necessary for maintaining strong intramolecular contacts (19). Given the role that these regions play in maintaining structural aspects of TcdB, we predicted that disrupting these molecular interactions could cause TcdB to adopt a conformation that prevents cellular intoxication.

Having identified a putative region of TcdB that influences toxin structure, we began to explore methods of targeting this region in order to destabilize the toxin and render it inactive. Here, we pursued an approach in which synthetic peptides based on sequences from TcdB2_1753–1851_ were designed and tested for TcdB-inhibitory activity. From this work, we identified 4 peptides with TcdB-inhibitory activity and then used a series of biophysical approaches to characterize one lead candidate peptide (PepB2). Experimental results indicate that PepB2 blocks TcdB toxicity by binding the CROP domain, forming a polymeric complex, and destabilizing the toxin.

## RESULTS

### Peptides based on sequences from amino acids 1753 to 1851 of TcdB2 inhibit cytotoxicity.

**FIG 1:**
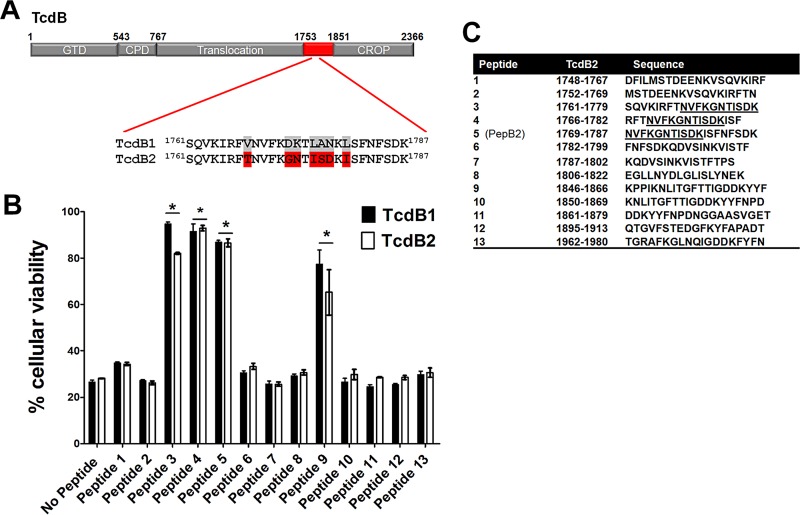
Screen identifying TcdB-inhibitory peptides. (A) Domain layout of TcdB highlighting the region where the screen for inhibitory peptides was focused (red segment of TcdB). This schematic also presents the sequence of TcdB2 that is rich in inhibitory peptides and provides a comparison of the same sequence in TcdB1. (B) Peptides from TcdB2 were synthesized and screened for inhibition of TcdB1 or TcdB2 activity in CHO-K1 cells. The bar graph represents the percent cellular viability after treatment for 24 h with 0.2 pM TcdB in the presence and absence of 50 µM peptide. Data are presented as means (*n* = 3) ± standard deviations. Asterisks indicate significant increases above toxin-treated controls. *, *P* < 0.001. (C) List of peptide sequences tested in this screen. The underlined sequence reveals a consensus sequence shared by 3 inhibitory peptides.

As shown in Fig. 1, a series of water-soluble peptides spanning amino acids 1753 to 1851 of TcdB2 (highlighted in Fig. 1A) were designed and tested for inhibitory activity. Each peptide was tested against TcdB1 and TcdB2 using CHO-K1 cells, which are highly sensitive to TcdB. In addition to the 1753-to-1851 peptides, 3 peptides (numbered 11 to 13) derived from the proximal 1851-to-2366 region of TcdB2 were included for comparison. This screen identified 4 inhibitory peptides. Three peptides were derived from the 1761-to-1787 region, and another inhibitory peptide with weaker activity was located at residues 1846 to 1866 (Fig. 1B). Each of the three peptides exhibiting the most potent inhibition included amino acids 1769 to 1779 (Fig. 1C), which contain a sequence that we previously identified as necessary for supporting intratoxin interactions in TcdB2 (19).

**FIG 2:**
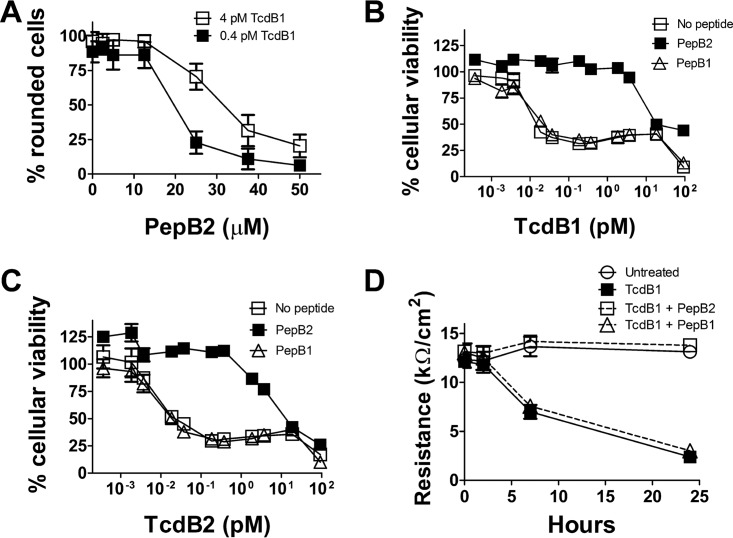
TcdB inhibition with a peptide derived from TcdB2 but not TcdB1. (A) Cell rounding assay examining different concentrations of PepB2 that inhibit 4 pM or 0.4 pM TcdB1. The graph represents the percentage of cells rounding after 2 h, and data are presented as the mean from 6 different fields ± standard deviation. (B and C) Cytotoxicity assay demonstrating that PepB2 protects CHO-K1 cells from a wide range of TcdB1 or TcdB2 concentrations. The graph represents the percent cellular viability after treatment for 24 h with TcdB in the presence and absence of 50 µM PepB2 or PepB1. Data are presented as means (*n* = 3) ± standard deviations. (D) PepB2 prevents TcdB-induced damage to tight junctions as shown by measuring transepithelial resistance in T84 cells. T84 cells were grown in a transwell plate until the formation of tight junctions and then exposed to 40 pM TcdB1 in the presence and absence of 50 µM PepB2 or PepB1. Electrical resistance values were then recorded at the indicated time points. Data are presented as means (*n* = 4) ± standard deviations. A decrease in resistance values indicates that tight junctions are disrupted.

The next set of experiments examined the inhibitory activity of the 19-amino-acid peptide spanning amino acids 1769 to 1787 of TcdB2 (termed PepB2). In the first experiment, the MIC of PepB2 was found to be between 25 μM and 50 μM when TcdB1 was incubated with CHO-K1 cells for 2 h in the presence of the peptide (Fig. 2A). Next, we examined the ability of PepB2 to provide long-term protection from TcdB1 and TcdB2 over a range of toxin concentrations (Fig. 2B and C). In these experiments, CHO-K1 cells were exposed to TcdB, plus or minus PepB2, and toxicity was quantified after 24 h. Results from this study demonstrated that PepB2 increased the 50% toxic concentration (TC_50_) of TcdB1 500-fold and the TC_50_ of TcdB2 200-fold (Fig. 2B and C). In addition to the inhibitory effects on CHO-K1 cells, PepB2 prevented TcdB-mediated disruption of transepithelial resistance (Fig. 2D) in a polarized human epithelial cell line (T84 cells). In contrast to PepB2, a peptide (PepB1) based on the 1769-to-1787 region of TcdB1 did not inhibit the cytotoxicity of TcdB1 or TcdB2 (Fig. 2B to D). In contrast to the inhibition of TcdB1 and TcdB2, PepB2 did not block cytotoxicity of *C. difficile* TcdA or *Bacillus anthracis* lethal toxin (see Fig. S1 in the supplemental material).

10.1128/mBio.00503-17.1 FIG S1  PepB2 does not inhibit TcdA or *Bacillus anthracis* lethal toxin (LT). (A) PepB2 does not reduce TcdA-induced cytotoxicity in CHO-K1 cells. The graph represents the percent cellular viability after treatment for 24 h with TcdA in the presence and absence of 50 µM PepB2 or PepB1. Data are presented as means (*n* = 3) ± standard deviations. (B) PepB2 does not reduce the cytotoxicity activity of LT in RAW 264.7 cells. The graph depicts the percent cellular viability after RAW 264.7 cells are exposed to LT for 24 h with 50 µM PepB2 or PepB1. Data are presented as means (*n* = 3) ± standard deviations. Download FIG S1, TIF file, 2 MB. Copyright © 2017 Larabee et al. 2017 Larabee et al. This content is distributed under the terms of the Creative Commons Attribution 4.0 International license.

### PepB2 does not inhibit TcdB enzymatic activities.

**FIG 3:**
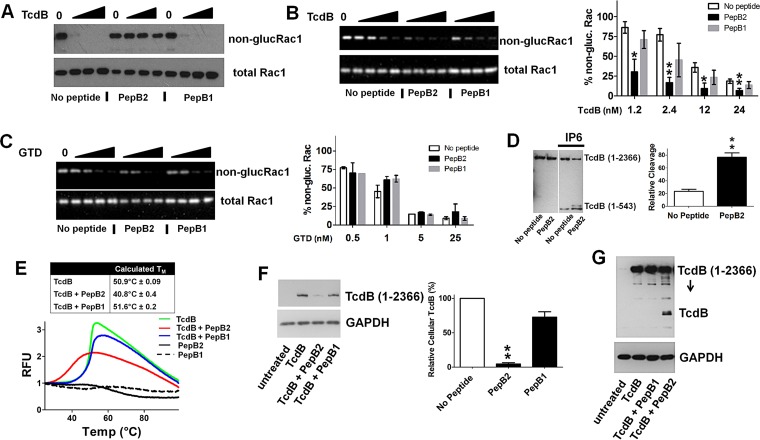
Impact of PepB2 on TcdB enzymatic activity, thermal stability, and cell association. (A) Immunoblot analysis of intracellular Rac1 glucosylation in CHO-K1 cells exposed to TcdB (0.037 pM, 0.37 pM, and 3.7 pM) for 25 h with and without PepB1 (50 µM) or PepB2 (50 µM). (B and C) *In vitro* Rac1 glucosylation assay. In these assays, purified Rac1 and UDP-glucose were combined with TcdB (1.2 nM, 2.4 nM, 12 nM, and 24 nM) (B) or GTD (0.5 nM, 1 nM, 5 nM, and 25 nM) (C). These experiments were carried out in the presence and absence of 50 µM PepB1 or PepB2, and Rac1 glucosylation was analyzed by immunoblotting. In the bar graph, the immunoblot data were quantified by densitometry analysis for TcdB (*n* = 3) and GTD (*n* = 2). (D) *In vitro* autoprocessing assay. TcdB autoprocessing was triggered by incubating TcdB (37 pM) for 1 h at 37°C with 500 µM IP6 in the presence and absence of 50 µM PepB1 or PepB2. Autoprocessing activity was evaluated by immunoblotting using an antibody that recognizes the amino-terminal domain of TcdB. Densitometry analysis was performed on the bands corresponding to the GTD cleaved from full-length TcdB (*n* = 4). (E) Differential scanning fluorimetry (DSF) was used to determine the thermal stability (*T*_*m*_) of TcdB (740 nM) with and without 500 µM PepB1 or PepB2. The graph depicts increases in relative fluorescence units (RFU) as SYPRO Orange binds to hydrophobic regions of proteins that undergo temperature-induced unfolding. From these data, the *T*_*m*_ was calculated and is displayed as mean (*n* = 4) and standard deviation. (F) Immunoblot analysis of TcdB associating with cells for 10 min. CHO-K1 cells were exposed at 37°C to 4 nM TcdB in the presence and absence of 50 µM peptide. The cells were then washed, and total cell lysates were examined by immunoblotting. The bar graph represents densitometry analysis of the immunoblot data (*n* = 4) from the 10-min exposure to TcdB. (G) Immunoblot analysis of TcdB associating with cells for 30 min. CHO-K1 cells were exposed at 37°C to 4 nM TcdB in the presence and absence of 50 µM peptide. The cells were then washed, and total cell lysates were examined by immunoblotting. In this figure, all bar graphs represent the mean densitometry value ± standard deviation, and asterisks indicate significant change. *, *P* < 0.01; **, *P* < 0.001.

In the final step of cellular intoxication, TcdB glucosylates small GTPases such as Rac1. Using an antibody that reacts only with nonglucosylated Rac1, the *in vivo* and *in vitro* glucosyltransferase activity of TcdB was measured in the presence and absence of PepB2. As shown in Fig. 3A, PepB2 prevented Rac1 glucosylation by TcdB in treated cells. In comparison, however, PepB2 did not suppress the *in vitro* glucosylation of Rac1 by TcdB but instead appeared to increase enzymatic activity (Fig. 3B). This did not appear to be the result of PepB2 targeting the glucosyltransferase domain (GTD) directly because the peptide had no effect on the glucosyltransferase activity of purified recombinant GTD (TcdB aa 1 to 543 [Fig. 3C]). The impact of PepB2 on TcdB autoprocessing was also examined using an *in vitro* assay in the presence of inositol hexakisphosphate 6 (IP6). Similarly to the effect on *in vitro* glucosylation, PepB2 enhanced the efficiency of IP6-induced TcdB autoproteolysis (Fig. 3D). These results suggest that PepB2 might increase susceptibility to enzymatic activity possibly by introducing conformational instability into TcdB. To determine if PepB2 alters TcdB structural integrity, thermal stability profiles were generated using differential scanning fluorimetry (DSF). As shown in Fig. 3E, the melting temperature (*T*_*m*_) for TcdB alone was found to be 50.9°C while the addition of PepB2 substantially reduced the *T*_*m*_ of TcdB to 40.8°C. Overall, these results suggest that PepB2 does not block TcdB enzymatic activities but could remove structural constraints that limit the enzymatic activities of TcdB.

### PepB2 disrupts the ability of TcdB to engage cells.

Because PepB2 did not inhibit the enzymatic activities of TcdB *in vitro*, we reasoned that PepB2 could alter how TcdB engages cells. To explore this, cells were exposed to TcdB for 10 or 30 min in the presence or absence of PepB2, and total cellular association was then measured by immunoblotting. Using this assay, we made two important observations. First, at the early time point, associations between TcdB and cells are significantly reduced in the presence of PepB2 but not in the presence of PepB1 (Fig. 3F). Second, with a longer incubation, TcdB interaction with cells is not reduced by PepB2; however, PepB2 causes TcdB to become susceptible to proteolytic cleavage (Fig. 3G). These data suggest that PepB2 slows the temporal course of TcdB binding to cells and increases toxin sensitivity to intracellular proteolytic cleavage.

### PepB2 multivalent interactions with the CROP region of TcdB.

**FIG 4:**
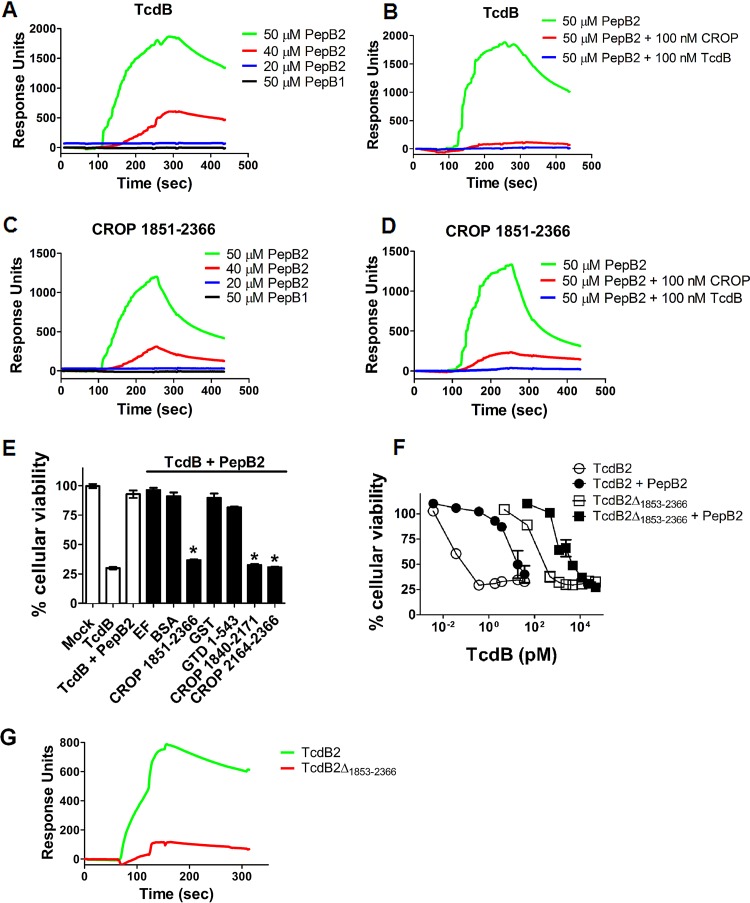
PepB2 binds TcdB at the CROP domain. (A to D) Surface plasmon resonance (SPR) analysis of PepB2 binding TcdB or the CROP domain (TcdB2_1851–2366_). TcdB2 or CROP domain was flow cell immobilized, and PepB2 or PepB1 was injected at a flow rate of 5 μl/min at 25°C. The sensorgrams presented are subtracted from a reference cell containing immobilized BSA. For the competition experiments, 100 nM TcdB2 or CROP domain was combined with PepB2 before injection. (E) Cytotoxicity activity of TcdB in the presence of PepB2 with proteins that absorb the peptide inhibitory activity. The bar graph shows the percent viability of CHO-K1 cells after treatment for 24 h with 0.75 pM TcdB in the presence of 50 µM PepB2. In this assay, PepB2-absorbing proteins were tested at a 1 µM concentration. Data are presented as means (*n* = 3) ± standard deviations. Asterisks indicate significant decreases in cytotoxicity compared to the TcdB-plus-PepB2 condition. *, *P* < 0.001. (F) Cytotoxicity assay comparing TcdB2 and TcdB2Δ_1853–2366_ in CHO-K1 cells. These graphs represent the percent cellular viability after treatment for 24 h. Data are presented as means (*n* = 3) ± standard deviations. (G) SPR analysis comparing binding of PepB2 to equal moles of TcdB2 and TcdB2Δ_1853–2366_. Toxins were flow cell immobilized, and 40 µM PepB2 was injected at a flow rate of 20 μl/min at 25°C.

Our previous work demonstrated that the PepB2 sequence within intact TcdB is necessary for preserving strong intramolecular contacts that influence the exposure of epitopes in the CROP region of the toxin (19). This observation led us to hypothesize that PepB2 binds to TcdB in a way that mimics the structural interactions mediated by the 1769-to-1787 region of TcdB. Thus, surface plasmon resonance (SPR) was used to characterize PepB2-TcdB interactions. In Fig. 4A, a series of sensorgrams reveals that PepB2, but not PepB1, binds TcdB conjugated to a sensor chip. PepB2 did not bind a bovine serum albumin (BSA)-coated reference channel. When we examined the change in response units as PepB2 bound immobilized TcdB, we calculated that approximately 250 molecules of PepB2 bound a single molecule of TcdB. The large number of PepB2 molecules binding TcdB was confirmed by the observation that 100 nM soluble TcdB was sufficient to block 50 μM PepB2 binding to sensor chip-immobilized TcdB. The large ratio of PepB2 to TcdB detected by SPR suggested that oligomers of PepB2 may be binding TcdB (see Fig. 6) and suggested that PepB2 could be targeting a repeating structure such as the CROP domain.

To determine if PepB2 targets the CROP domain, experiments were designed to test if PepB2 binding to TcdB could be inhibited in the presence of purified CROP domain (TcdB2_1851–2366_). As shown in Fig. 4B, the sensorgram reveals that the CROP domain blocks binding between PepB2 and TcdB. We also conjugated TcdB2_1851–2366_ to the sensor chip and obtained results that indicated a similar binding mechanism as observed with full-length TcdB (Fig. 4C and D). Since PepB2 appeared to interact with the CROP region of TcdB, we next tested whether adding purified CROP to a cytotoxicity assay could counteract the TcdB-inhibitory activity of PepB2. In control experiments, purified CROP (TcdB2_1851–2366_) did not cause cytotoxicity and did not alter the level of TcdB-induced cytotoxicity. However, TcdB2_1851–2366_ blocked PepB2 inhibition of TcdB (Fig. 4E). Interestingly, the amount of CROP (1 μM) needed to block PepB2 activity was far below the molar concentration of PepB2 (50 μM), providing further evidence of a multivalent interaction between PepB2 and the CROP domain. Further supporting the specificity of this interaction, we were unable to compete away PepB2 inhibitory activity with BSA, glutathione *S*-transferase (GST), *Bacillus anthracis* edema factor (EF), or purified GST-tagged GTD (Fig. 4E). To determine if PepB2 binds a distinct region of the CROP domain, we tested GST-tagged forms of shorter CROP sections such as TcdB2_1840–2171_ (14 repeats) and TcdB2_2164–2366_ (8 repeats). As shown in Fig. 4E, both TcdB2_1840–2171_ and TcdB2_2164–2366_ blocked PepB2 inhibitory activity, suggesting that multiple sections of the CROP domain are able to bind PepB2. As a last test of CROP dependence, we examined the PepB2 sensitivity of a form of TcdB2 that is truncated at amino acid 1853 (TcdB2Δ_1853–2366_). This truncated toxin has all but two CROP repeats removed and is 1,000-fold less cytotoxic than full-length TcdB2 (Fig. 4F). As shown in Fig. 4F, TcdB2Δ_1853–2366_ was 10-fold less sensitive to PepB2 than full-length TcdB2. To support these cytotoxicity results, levels of PepB2 binding to equal molar amounts of TcdB2 and TcdB2Δ_1853–2366_ were compared using SPR. Results from this analysis revealed that much higher levels of PepB2 bound TcdB2 than TcdB2Δ_1853–2366_ (Fig. 4G), which correlated with the cytotoxicity data.

### The GN_5-6_ amino acid pair in PepB2 is critical for structure and function.

**FIG 5:**
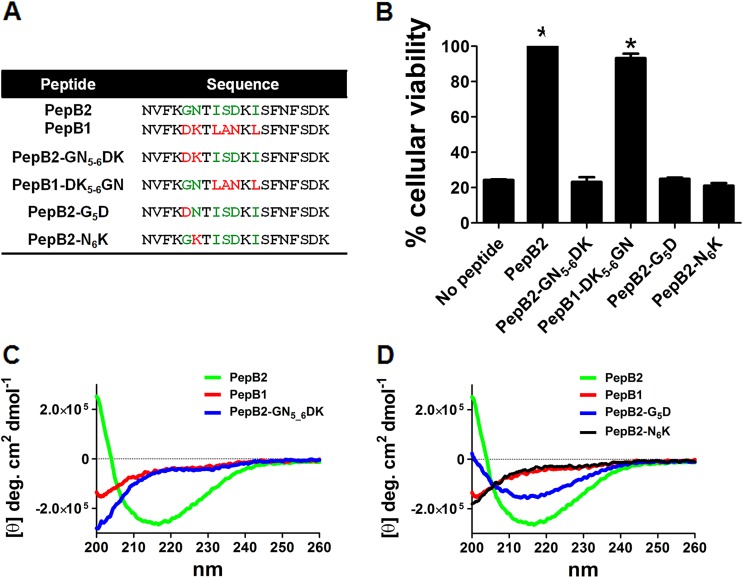
Identification of PepB2 residues necessary for inhibiting TcdB. (A) Sequence alignment comparing PepB1, PepB2, and variant peptides. Green residues are found only in PepB2, red residues are found only in PepB1, and black residues are found in both peptides. (B) Comparison of the TcdB-inhibitory activities of the variant peptides in CHO-K1 cells. The bar graph shows the percent cellular viability after treatment for 24 h with 0.4 pM TcdB1 in the presence of 50 µM (each) variant peptides. Data are presented as means (*n* = 3) ± standard deviations. Asterisks indicate significant increases above toxin-treated controls. *, *P* < 0.001. (C and D) Comparisons of CD spectra from PepB1, PepB2, and variant peptides described in panel A. The *y* axis is presented in molar ellipticity [θ]. CD spectra were obtained with 40 μM peptide in PBS at 25°C.

To help dissect PepB2 structural elements, experiments were designed to uncover key amino acids in PepB2 necessary for the inhibition of TcdB. To guide these experiments, we took advantage of sequence differences between the inhibitory PepB2 and the noninhibitory PepB1 that is unable to bind TcdB (alignment in Fig. 5A). A series of peptides were designed in which amino acids that differ between PepB2 and PepB1 were substituted to reflect those found in the reciprocal peptide (Fig. 5A). These variant PepB2 peptides were then tested for their ability to inhibit TcdB cytotoxicity (Fig. 5B). As shown in Fig. 5B, when GN_5-6_ from PepB2 was replaced with DK_5-6_ from PepB1 (PepB2-GN_5-6_DK), the resulting peptide no longer inhibited the toxin. Conversely, inhibitory activity was gained in PepB1 by the reciprocal substitution producing PepB1-DK_5-6_GN (Fig. 5B). To determine if G_5_ is necessary for the activity of PepB2, we replaced G_5_ with the D_5_ from PepB1 (PepB2-G_5_D) and found that this single substitution was sufficient to abrogate inhibitory activity (Fig. 5B). We also replaced N_6_ in PepB2 with K_6_ from PepB1 (PepB2-N_6_K) and found that TcdB-inhibitory activity disappeared (Fig. 5B).

Since the GN_5-6_ pair in PepB2 appeared to be crucial for toxin inhibition, we next determined if the GN_5-6_ pair was required for PepB2 to form an organized structure. Thus, circular dichroism (CD) analysis was utilized to gain structural insight into PepB2 and determine if the GN_5-6_-to-DK_5-6_ switch resulted in an altered peptide structure. As shown in Fig. 5C, the CD spectra of PepB1 and PepB2 were markedly different. PepB1 appeared to be unstructured, while PepB2 adopted an ordered structure that produces a CD spectrum with a minimum near 217 nm, suggesting a significant level of β-sheet. Analysis of PepB2-GN_5-6_DK revealed a CD spectrum similar to that of PepB1 and not PepB2 (Fig. 5C). Thus, PepB2-GN_5-6_DK is disordered and the GN_5-6_ pair is critical for PepB2 adopting an ordered structure. Further examination of the GN pair in PepB2 revealed that switching the G_5_ with D_5_ (PepB2-G_5_D) resulted in a CD spectrum showing some structural organization but lacking the PepB2 conformation (Fig. 5D). Replacing N_6_ with K_6_ in PepB2 (PepB2-N_6_K) resulted in a disordered conformation resembling PepB1. As shown in the supplemental material, other variant forms of PepB2 were examined, and the results revealed that TcdB-inhibitory activity was dramatically reduced or completely lost when the length of PepB2 was slightly reduced (Fig. S2) or when phenylalanines were exchanged for alanines, tyrosines, or tryptophans (Fig. S3). CD analysis of these variant peptides (Fig. S2 and S3) also demonstrated a correlation between TcdB-inhibitory activity and peptide conformation.

10.1128/mBio.00503-17.2 FIG S2  Activity and conformation of truncated PepB2. (A) Sequence alignment comparing truncated forms of PepB2 examined in these experiments. (B) Comparison of the TcdB-inhibitory activity of truncated PepB2 in CHO-K1 cells. The bar graph shows the percent cellular viability after treatment for 24 h with 0.4 pM TcdB1 in the presence of 50 µM (each) peptide. Data are presented as means (*n* = 3) ± standard deviations. Asterisks indicate significant increases above toxin-treated controls. *, *P* < 0.001. (C) Comparison of CD spectra from PepB2 and truncated forms. Download FIG S2, TIF file, 2.9 MB. Copyright © 2017 Larabee et al. 2017 Larabee et al. This content is distributed under the terms of the Creative Commons Attribution 4.0 International license.

10.1128/mBio.00503-17.3 FIG S3  Phenylalanines are necessary for PepB2 activity. (A) PepB2 sequence denoting location of phenylalanines and the sequence of variant peptides where phenylalanines are replaced. (B) A comparison of the TcdB-inhibitory activity of PepB2 and phenylalanine variant peptides in CHO-K1 cells. The bar graph shows the percent cellular viability after treatment for 24 h with 0.04 pM TcdB1 in the presence of 50 µM each peptide. Data are presented as means (*n* = 3) ± standard deviations. Asterisks indicate significant increases above toxin-treated controls. *, *P* < 0.001. (C) Comparison of CD spectra from PepB2 and phenylalanine variant peptides. Download FIG S3, TIF file, 2.8 MB. Copyright © 2017 Larabee et al. 2017 Larabee et al. This content is distributed under the terms of the Creative Commons Attribution 4.0 International license.

### PepB2 forms an oligomer.

**FIG 6:**
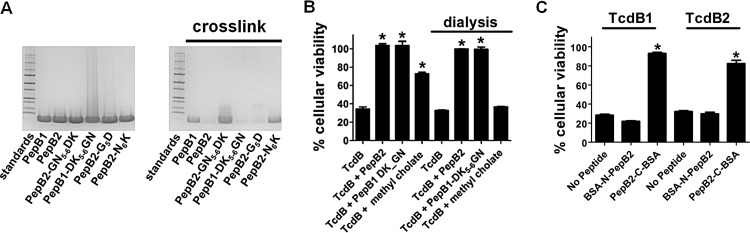
PepB2 functions as an oligomer and is enhanced when conjugated to albumin. (A) Comparison of oligomerization of PepB1, PepB2, and variant peptides described in Fig. 5A. Peptide oligomers were detected by exposing peptides to a cross-linking agent, BS^3^. The resulting cross-linked peptides were then analyzed by 4 to 20% gradient SDS-PAGE and stained with Coomassie blue. (B) PepB2, PepB1-DK_5-6_GN, and methyl cholate were placed in serum-free medium and dialyzed using 50-kDa-cutoff membranes. After extensive dialysis, the molecules contained within the dialysis tube were tested for TcdB-inhibitory activity in a cytotoxicity assay. The bar graph shows the percent viability of CHO-K1 cells after treatment for 24 h with 0.4 pM TcdB in the presence of either controls, nondialyzed inhibitor, or dialyzed inhibitor. Data are presented as means (*n* = 3) ± standard deviations. Asterisks indicate significant increases above toxin-treated controls. *, *P* < 0.001. (C) TcdB-inhibitory activity of 1.5 µM BSA-N-PepB2 or 1.5 µM PepB2-C-BSA in CHO-K1 cells. The bar graph indicates percent viability of cells after treatment for 24 h with 0.2 pM TcdB1 or TcdB2. Data are presented as means (*n* = 3) ± standard deviations. Asterisks indicate significant increases above toxin-treated controls. *, *P* < 0.001.

The SPR data from Fig. 4 suggest that PepB2 may be binding TcdB as an oligomer; thus, we examined this possibility in the next series of experiments. In preliminary work, mass spectrometry (MS) analysis confirmed the expected mass of monomeric PepB2 (2,161.38 Da) when analyzed in the presence of organic solvents. However, when PepB2 was placed in buffer resembling physiological conditions, we observed PepB2 oligomers (Fig. 6). As shown in Fig. 6A, treatment of PepB2 with the amine-reactive cross-linker bis(sulfosuccinimidyl)suberate (BS^3^) resulted in a form of PepB2 unable to migrate by SDS-PAGE (Fig. 6A). In contrast, cross-linked PepB1 migrated into the gel in a similar pattern as uncross-linked control peptides (Fig. 6A). Variant peptides from Fig. 5 were also cross-linked and analyzed. As shown in Fig. 6A, PepB2 is unable to oligomerize when the GN_5-6_ is replaced with DK_5-6_ (PepB2-GN_5-6_DK), and PepB1 gains the ability to oligomerize when DK_5-6_ is switched with GN_5-6_ (PepB1-DK_5-6_GN). Further evaluation of the GN_5-6_ pair revealed that replacing G_5_ with D_5_ (PepB2-G_5_D) did not abolish oligomerization, but swapping N_6_ with K_6_ (PepB2-N_6_K) resulted in loss of oligomerization (Fig. 6A). These data support the idea that PepB2 forms oligomers and reveal that N_6_ influences the ability of this peptide to oligomerize.

Next, we determined if the oligomeric form of PepB2 possesses TcdB-inhibitory activity. In these experiments, PepB2 was extensively dialyzed using 50-kDa-cutoff dialysis membranes, and the molecules retained within the dialysis tube were then tested for TcdB-inhibitory activity. As shown in Fig. 6B, TcdB-inhibitory activity was not removed when PepB2 was dialyzed, demonstrating that inhibitory PepB2 was forming an oligomer larger than 50 kDa. We also dialyzed the variant PepB1 peptide (PepB1-DK_5-6_GN) that gains TcdB-inhibitory activity when DK_5-6_ is replaced with GN_5-6_ (Fig. 5). These results revealed that TcdB-inhibitory activity from PepB1-DK_5-6_GN was not removed by dialysis (Fig. 6B). As a control in Fig. 6B, methyl cholate (422.6 Da), a small-molecule TcdB inhibitor (20), was also subjected to dialysis, and the toxin-inhibitory activity was lost.

### PepB2 is enhanced by BSA conjugation.

Because the bulky nature of oligomeric PepB2 may be necessary to inactivate TcdB, the function of PepB2 may be improved by conjugating the peptide to a large molecule such as albumin. Furthermore, albumin is an established drug carrier that is utilized by several peptide-based drugs that are currently approved for clinical usage (21, 22). To this end, PepB2 was chemically attached to bovine serum albumin (BSA) either through the amino-terminal domain of PepB2 (BSA-N-PepB2) or through the carboxyl-terminal domain of PepB2 (PepB2-C-BSA). As shown in Fig. 6C, BSA-N-PepB2 did not protect cells from TcdB cytotoxicity; however, PepB2-C-BSA was able to inhibit TcdB-induced cytotoxicity. Moreover, BSA conjugation to the carboxyl terminus of PepB2 appeared to improve PepB2 effectiveness because PepB2-C-BSA inhibited TcdB at approximately 1.5 μM (Fig. 6C) while nonderivatized PepB2 inhibited in the range of 25 to 50 μM (Fig. 2).

## DISCUSSION

Data from this work support three important conclusions. First, TcdB can be deactivated using 17- to 20-amino-acid peptides derived from a region of the toxin that is necessary for forming intramolecular contacts. Second, TcdB is inhibited by PepB2 through a mechanism that depends on CROP domain targeting that results in toxin destabilization and delayed cell binding. Third, inhibition strongly correlates with the ability of PepB2 to oligomerize and adopt an organized conformation. Taken together, these findings reveal a class of antitoxin peptides that inhibit a critical *C. difficile* virulence factor.

The concept of developing peptide-based inhibitors against bacterial toxins is not unprecedented. For instance, Abdeen et al. enriched for random peptides that inhibit TcdA glucosyltransferase activity (23). Also, using a phage display enrichment approach, Mourez et al. identified a peptide with low affinity to the anthrax protective antigen heptamer and designed a polyvalent form of the peptide that blocked anthrax toxin *in vivo* (24). However, our approach differs substantially from these screening and enrichment strategies. In contrast to those studies, we selected candidate peptides based on biochemical data pointing to the 1753-to-1851 region as a promising target in TcdB. Whether this approach to selecting peptide-based inhibitors will be applicable to other multidomain toxins or if this effect is unique to TcdB remains to be determined. However, because many intracellular bacterial toxins must undergo large synchronized structural rearrangements and insert into membranes, these proteins are predicted to be inherently unstable (25). Thus, 17- to 20-amino-acid peptides that mimic elements in the toxin necessary for forming intramolecular contacts may provide enough destabilization that the toxin becomes deactivated.

The data support the notion that PepB2 inhibits TcdB by causing structural changes to the toxin that disrupt steps in the intoxication process. As shown in Fig. 3, PepB2 reduced the *T*_*m*_ of TcdB and enhanced enzymatic activities known to be structurally constrained in large clostridial toxins (17, 26, 27). As a result of the PepB2-mediated changes to the TcdB structure, the toxin exhibited lower rates of cell binding and increased susceptibility to cell-associated proteolytic activity (Fig. 3). PepB2-associated changes in TcdB binding to cells are not surprising considering that the CROP domains influence the extent of TcdB cytotoxicity (Fig. 4F). The ability of TcdB to bind cells after longer exposures is also not unexpected, because receptors such as Frizzled bind TcdB by utilizing regions outside the CROP domain (5). Therefore, PepB2 may inhibit TcdB by changing the dynamics of cellular association, but we suspect that this is only one part of the overall inhibitory effect. Our observation that PepB2 causes increased susceptibility to cell-associated proteolytic activity suggests the peptide could invoke conformational changes that expose protease-sensitive sites in the toxin. Alternatively, PepB2-induced conformational instability may prevent the toxin from executing structural transitions necessary for membrane translocation during endocytosis and consequently result in extended exposure time to vesicular proteases.

The high ratio of PepB2 to TcdB detected by SPR binding experiments is indicative of multiple binding sites and perhaps other higher-order interactions between the peptide and toxin. Given the repeat sequence characteristics of the CROP domain, we suspected that this region could display multiple PepB2 binding sites. This idea of CROP domain targeting was verified by our SPR experiments (Fig. 4) and further reinforced using a cell-based competition experiment (Fig. 4E). This cell-based assay also corroborated the multivalent binding observed with SPR because the amount of CROP domain (1 μM) required to block PepB2 activity is far below the molar concentration of PepB2 (50 μM). This assay also demonstrated that multiple regions of the CROP domain (TcdB2_1840–2171_ and TcdB2_2164–2366_) are capable of binding PepB2 (Fig. 4E). However, this experiment did not demonstrate if all CROP repeats must bind PepB2 or if only a certain subset of CROP repeats must bind PepB2 in order for toxin inhibition to occur. We did begin to address this issue using a truncated form of TcdB (TcdB2Δ_1853–2366_) that retained 2 CROP repeats of a total of 25 repeats. TcdB2Δ_1853–2366_ was found to be 10-fold less sensitive to PepB2 inhibition than full-length TcdB2 (Fig. 4F). This decrease in sensitivity to PepB2 inhibition also correlated with the SPR data demonstrating that PepB2 bound TcdB2 much better than TcdB2Δ_1853–2366_ (Fig. 4G). Thus, two CROP repeats are sufficient to retain some toxin activity and remain sensitive to PepB2. While the presence of the complete CROP region significantly impacts PepB2 binding and this peptide can clearly bind to CROP sequences, we cannot yet rule out that PepB2 might also bind to other TcdB regions. This possibility could not be tested because highly purified recombinant TcdB with the entire CROP region deleted could not be obtained.

Evaluation of variant peptides revealed that the GN pair in PepB2 is necessary for the formation of an ordered structure as well as the inhibitory activity of the peptide. Because glycines as well as asparagines are commonly found in turn motifs (28–30), the GN pair may be stabilizing a turn motif in PepB2. CD analysis of PepB2 suggests that the peptide contains a significant level of β-sheet due to the occurrence of a 217-nm minimum in the spectrum. The CD data together with the essential nature of the GN pair suggest that PepB2 may be organizing into a β-hairpin structure. Further evaluation of the individual residues in the GN pair demonstrates that N_6_ is needed for oligomerization while G_5_ is dispensable (Fig. 6A). The CD data suggest that N_6_ makes a more substantial contribution to the PepB2 structure than G_5_ (Fig. 5D); however, both N_6_ and G_5_ are necessary for PepB2 to form an inhibitory peptide (Fig. 5B). Taken together, the ability to form oligomers combined with the ability to adopt a specific structure is required for PepB2 to inhibit TcdB. Therefore, oligomerization appears to be necessary for PepB2 function but is not a sole determinant of a TcdB-inhibiting peptide.

The ability of PepB2 to form oligomers is not unexpected considering that the sequence comprising PepB2 (TcdB2_1769–1787_) was previously found to support multimerization when evaluated within the isolated carboxyl-terminal domain of TcdB2 (19). As shown by chemical cross-linking/SDS-PAGE, PepB2 forms high-molecular-weight oligomers under physiological conditions while PepB1 does not (Fig. 6A). Results from the dialysis experiments also provide evidence that PepB2 oligomerization is necessary for TcdB inhibition (Fig. 6B). Thus, we postulated that large oligomers are needed to facilitate toxin destabilization and that attaching PepB2 to a large protein could potentially improve toxin inhibition. To test this idea, we conjugated PepB2 to albumin and found that this PepB2 modification reduced the molar amount required to inhibit TcdB (Fig. 6C). Indeed, formation of a polymeric structure may be a fortuitous characteristic of PepB2 that allows the peptide to not only interact with contact regions but also insert a molecule of sufficient size to sterically disrupt the TcdB structure and prevent the toxin from executing the steps required for cell entry.

## MATERIALS AND METHODS

### Toxins and peptides.

Peptides used in this study were synthesized and purified to 90% by GenScript (Piscataway, NJ). Peptide quality was assessed by high-performance liquid chromatography (HPLC) and mass spectrometry (MS). The theoretical and observed mass did not differ by more than 0.3 Da for any peptide used in this study. Conjugation of the amino-terminal domain of PepB2 to BSA was performed using glutaraldehyde, and conjugating the carboxyl-terminal domain of PepB2 to BSA was achieved using EDC [1-ethyl-3-(-3-dimethylaminopropyl)carbodiimide hydrochloride]. SDS-PAGE was employed to demonstrate that PepB2 was conjugated to BSA.

As previously described, recombinant TcdB1 (pC-His1622-TcdB1; gift from B. Lacy) and TcdB2 (pC-His1622-TcdB2) were produced in a *Bacillus megaterium* system (MoBiTec, Göttingen, Germany) and affinity purified by Ni^2+^ chromatography (19). TcdB2 mutants and truncations were generated in pC-His1622-TcdB2 using the QuikChange II XL site-directed mutagenesis kit (Agilent). The CROP domain (TcdB2_1851–2366_), smaller CROP domain fragments (TcdB2_1840–2171_ and TcdB2_2164–2366_), and the GTD (TcdB2_1–543_ and TcdB1_1–543_) were cloned from the *tcdB* gene (codon optimized for *Escherichia coli*) into the pET61Dest vector or pDest15 vector. The pET61Dest vector allowed for protein expression with an amino-terminal Strep-His tag in *E. coli*. The pDest15 vector permitted protein expression with an amino-terminal GST tag in *E. coli*. These expression plasmids were transformed into *E. coli* BL21 Star(DE3), and the resulting cultures were grown to an *A*_600_ of 0.8. Then, these cultures were induced with 300 μM isopropyl-1-thio-β-d-galactopyranoside for 16 h at 16°C, and protein was purified by affinity chromatography (19). Recombinant TcdA was obtained as a gift from B. Lacy. *Bacillus anthracis* lethal factor, edema factor, and protective antigen were purchased from List Biological Laboratories.

### Cell rounding and cytotoxicity assays.

CHO-K1 cells (ATCC) were cultured at 37°C in the presence of 6% CO_2_ with F-12–K medium containing 10% fetal bovine serum (FBS), 100 units/ml penicillin, and 100 μg/ml streptomycin. For cell rounding assays, the cells were seeded in 24-well plates at a density of 5.0 × 10^4^ cells/well and allowed to attach overnight. On the next day, TcdB with and without PepB2 was diluted into complete culture medium and then added to cells. Cells were incubated with toxin for 2 h and then fixed for 10 min with phosphate-buffered saline (PBS) containing 4% formaldehyde. After imaging fixed cells, both nonrounded and rounded cells were counted. For each data point, approximately 200 cells were counted from 6 fields taken from 2 separate wells.

In cytotoxicity assays, CHO-K1 cells were seeded in 96-well plates at a density of 1.0 × 10^4^ cells/well and allowed to attach overnight. Cells were incubated with toxin or toxin plus peptide for 24 h, and then cell viability was quantified by the cell counting kit 8 (CCK-8) assay (Sigma), a WST-8 dye-based colorimetric reaction.

### TER.

Transepithelial electrical resistance (TER) was measured in a human carcinoma cell line (T84; ATCC). T84 cells were cultured in medium consisting of a 1:1 mixture of F-12–K medium (ATCC) and Dulbecco’s modified Eagle’s medium (DMEM) (ATCC; modified with 4 mM l-glutamine, 4,500 mg/liter glucose, 1 mM sodium pyruvate, and 1,500 mg/liter sodium bicarbonate). This 1:1 mixture of culture media also included 5% FBS, 100 units/ml penicillin, and 100 μg/ml streptomycin. T84 cells were seeded at a density of 6.0 × 10^4^ cells/well in a transwell plate with a growth area of 0.3 cm^2^ and a membrane composed of tissue culture-treated polyethylene terephthalate with a pore size of 3 µm. These cells were cultured until tight junctions formed (about 1 week). Electrical resistance values for the T84 cells were then recorded at the experimental time points indicated on the graph in Fig. 2D.

### Analysis of intracellular Rac1 glucosylation.

CHO-K1 cells were cultured as described above and were seeded the day before the experiment in 24-well plates at a density of 5.0 × 10^4^ cells/well. TcdB was then combined with peptide in complete culture medium and added to cells. Following this exposure, total proteins were extracted by removing culture medium and adding cold lysis buffer (1% SDS, 50 mM Tris-HCl [pH 7.4], 5 mM EDTA, and a protease inhibitor mixture). For each condition, equal amounts of total protein were analyzed by immunoblot probing with a mouse monoclonal antibody to total Rac1 (EMD Millipore; catalog no. 05-389) or a mouse monoclonal antibody recognizing nonglucosylated Rac1 (BD Bioscience; catalog no. 610651).

### Analysis of TcdB associating with cells.

CHO-K1 cells were cultured as described above and were seeded the day before the experiment in 12-well plates at a density of 1.0 × 10^5^ cells/well. These cells were then exposed for 10 min or 30 min at 37°C to 4 nM of TcdB in the presence and absence of peptide. After this exposure, the cells were then washed twice with 1 ml of complete medium, and then total proteins were extracted by adding cold lysis buffer (1% SDS, 50 mM Tris-HCl [pH 7.4], 5 mM EDTA, and a protease inhibitor mixture). For each condition, equal amounts of total protein were analyzed by immunoblot probing with antibodies to the amino-terminal domain of TcdB (R&D Systems; catalog no. AF6246) or an antibody to glyceraldehyde-3-phosphate dehydrogenase (GAPDH) (Abcam; catalog no. AB8245).

### *In vitro* glucosylation assay.

To determine how PepB2 influences the glucosylation activity of TcdB, 400 nM purified GST-Rac1 and 40 μM UDP-glucose were combined with a range of concentrations of TcdB (1.2 nM to 24 nM) or GTD (0.5 nM to 25 nM). These reactions were performed in 20 μl of buffer consisting of 50 mM HEPES (pH 7.5), 100 mM KCl, 2 mM MgCl_2_, 1 mM MnCl_2_, and 100 μg/ml BSA. The reactions were carried out at 37°C for 1 h and were stopped by heating the samples at 95°C for 7 min in sample buffer (62.5 mM Tris-HCl [pH 6.8], 2% SDS, 10% glycerol, 5% β-mercaptoethanol, and 0.001% bromphenol blue). An immunoblotting assay was then performed by probing with a mouse monoclonal antibody to total Rac1 (EMD Millipore; catalog no. 05-389) or a mouse monoclonal antibody recognizing nonglucosylated Rac1 (BD Bioscience; catalog no. 610651).

### *In vitro* autoprocessing assay.

TcdB autoprocessing was activated by incubating 37 pM TcdB with 500 µM inositol hexakisphosphate (IP6) for 1 h at 37°C in a buffer consisting of 50 mM HEPES (pH 7.5), 150 mM NaCl, and 20 mM dithiothreitol (DTT). After the reaction was stopped by heating the samples at 95°C for 7 min in SDS-PAGE sample buffer, cleavage of TcdB was evaluated by an immunoblot assay probing with antibodies to the amino-terminal domain of TcdB (R&D Systems; catalog no. AF6246).

### DSF.

The thermal stability (*T*_*m*_) was determined by combining purified TcdB with SYPRO Orange dye and then monitoring fluorescence emissions as dye bound to hydrophobic regions of proteins that became exposed during temperature-induced unfolding. An Applied Biosystems 7500 real-time PCR system was used to monitor fluorescence emission as temperature was stepped from 25°C to 99°C. These reactions were performed in quadruplicate in a buffer consisting of 20 mM HEPES (pH 8.0) and 150 mM NaCl. *T*_*m*_ values are the temperatures at the midpoint of the transition from folded to unfolded and are found by plotting the first derivative of fluorescence versus temperature.

### SPR.

Surface plasmon resonance (SPR) analysis was performed using a Biacore 3000 instrument (OMRF Biacore Core Facility). TcdB2, TcdB2Δ_1853–2366_, TcdB2_1851–2366_, or BSA was captured on a CM5 sensor chip via amine coupling chemistry based on EDC/*N*-hydroxysuccinimide (NHS) activation of the carboxymethyl groups of the sensor chip surface. For TcdB2 conjugation, 1 μM TcdB2 or TcdB2Δ_1853–2366_ was injected at a flow rate of 10 μl/min. TcdB2_1851–2366_ was injected at a concentration of 2.5 μM with a flow rate of 10 μl/min. After protein conjugation, ethanolamine was passed over the sensor chip to deactivate unreacted NHS esters. The running buffer used for the binding experiments consisted of 25 mM HEPES (pH 7.4), 150 mM NaCl, and 0.01% Tween 20, and the chip regeneration buffer consisted of 50 mM NaOH and 1 M NaCl.

### CD.

The circular dichroism (CD) spectra of these peptides were recorded on a Jasco J715 spectropolarimeter (Jasco Corp., Tokyo, Japan) in a cuvette with a path length of 0.1 cm. Each spectrum was recorded at a wavelength range of 200 to 260 nm at 0.1-nm intervals with 3 accumulations per spectrum.

### Cross-linking.

Peptides were cross-linked using bis(sulfosuccinimidyl)suberate (BS^3^), which is a water-soluble homobifunctional cross-linking agent that reacts with primary amine groups. The cross-linking reaction was performed with 1.4 mM peptide and 5 mM BS^3^ in a buffer consisting of 25 mM HEPES (pH 7.4) and 150 mM NaCl. The reaction proceeded for 30 min at 25°C and then was quenched with 100 mM Tris, pH 8.0.

### Dialysis.

Inhibitors were diluted to 125 μM in F-12–K medium lacking FBS. Then, 1 ml of the diluted inhibitor was dialyzed with a 50-kDa-cutoff membrane into 2 liters of PBS while stirring. After 3 h, the PBS was discarded and replaced with an additional 1.5 liters of PBS. After 2 more hours of stirring dialysis, the inhibitors were removed from the dialysis tube and diluted into F-12–K medium containing 20% FBS. This produced an inhibitor concentration of approximately 62.5 μM that was then tested for TcdB inhibition using a cytotoxicity assay described above. As controls, a portion of the diluted inhibitors were subjected to mock dialysis conditions and also tested in a cytotoxicity assay.

## References

[1] Hunt JJ, Ballard JD 2013 Variations in virulence and molecular biology among emerging strains of Clostridium difficile. Microbiol Mol Biol Rev 77:567–581. doi:10.1128/MMBR.00017-13. 24296572 PMC3973386

[2] LaFrance ME, Farrow MA, Chandrasekaran R, Sheng J, Rubin DH, Lacy DB 2015 Identification of an epithelial cell receptor responsible for Clostridium difficile TcdB-induced cytotoxicity. Proc Natl Acad Sci U S A 112:7073–7078. doi:10.1073/pnas.1500791112. 26038560 PMC4460460

[3] Yuan P, Zhang H, Cai C, Zhu S, Zhou Y, Yang X, He R, Li C, Guo S, Li S, Huang T, Perez-Cordon G, Feng H, Wei W 2015 Chondroitin sulfate proteoglycan 4 functions as the cellular receptor for Clostridium difficile toxin B. Cell Res 25:157–168. doi:10.1038/cr.2014.169. 25547119 PMC4650570

[4] Pruitt RN, Lacy DB 2012 Toward a structural understanding of Clostridium difficile toxins A and B. Front Cell Infect Microbiol 2:28. doi:10.3389/fcimb.2012.00028. 22919620 PMC3417631

[5] Tao L, Zhang J, Meraner P, Tovaglieri A, Wu X, Gerhard R, Zhang X, Stallcup WB, Miao J, He X, Hurdle JG, Breault DT, Brass AL, Dong M 2016 Frizzled proteins are colonic epithelial receptors for C. difficile toxin B. Nature 538:350–355. doi:10.1038/nature19799. 27680706 PMC5519134

[6] Pruitt RN, Chambers MG, Ng KK, Ohi MD, Lacy DB 2010 Structural organization of the functional domains of *Clostridium difficile* toxins A and B. Proc Natl Acad Sci U S A 107:13467–13472. doi:10.1073/pnas.1002199107. 20624955 PMC2922184

[7] Albesa-Jové D, Bertrand T, Carpenter EP, Swain GV, Lim J, Zhang J, Haire LF, Vasisht N, Braun V, Lange A, von Eichel-Streiber C, Svergun DI, Fairweather NF, Brown KA 2010 Four distinct structural domains in Clostridium difficile toxin B visualized using SAXS. J Mol Biol 396:1260–1270. doi:10.1016/j.jmb.2010.01.012. 20070948

[8] Genisyuerek S, Papatheodorou P, Guttenberg G, Schubert R, Benz R, Aktories K 2011 Structural determinants for membrane insertion, pore formation and translocation of Clostridium difficile toxin B. Mol Microbiol 79:1643–1654. doi:10.1111/j.1365-2958.2011.07549.x. 21231971

[9] Zhang Z, Park M, Tam J, Auger A, Beilhartz GL, Lacy DB, Melnyk RA 2014 Translocation domain mutations affecting cellular toxicity identify the Clostridium difficile toxin B pore. Proc Natl Acad Sci U S A 111:3721–3726. doi:10.1073/pnas.1400680111. 24567384 PMC3956163

[10] von Eichel-Streiber C, Sauerborn M, Kuramitsu HK 1992 Evidence for a modular structure of the homologous repetitive C-terminal carbohydrate-binding sites of Clostridium difficile toxins and Streptococcus mutans glucosyltransferases. J Bacteriol 174:6707–6710. doi:10.1128/jb.174.20.6707-6710.1992. 1307487 PMC207659

[11] Hofmann F, Busch C, Prepens U, Just I, Aktories K 1997 Localization of the glucosyltransferase activity of Clostridium difficile toxin B to the N-terminal part of the holotoxin. J Biol Chem 272:11074–11078. doi:10.1074/jbc.272.17.11074. 9111001

[12] Egerer M, Giesemann T, Jank T, Satchell KJF, Aktories K 2007 Auto-catalytic cleavage of Clostridium difficile toxins A and B depends on cysteine protease activity. J Biol Chem 282:25314–25321. doi:10.1074/jbc.M703062200. 17591770

[13] Shen A, Lupardus PJ, Gersch MM, Puri AW, Albrow VE, Garcia KC, Bogyo M 2011 Defining an allosteric circuit in the cysteine protease domain of Clostridium difficile toxins. Nat Struct Mol Biol 18:364–371. doi:10.1038/nsmb.1990. 21317893 PMC3076311

[14] Zhang Y, Shi L, Li S, Yang Z, Standley C, Yang Z, ZhuGe R, Savidge T, Wang X, Feng H 2013 A segment of 97 amino acids within the translocation domain of Clostridium difficile toxin B is essential for toxicity. PLoS One 8:e58634. doi:10.1371/journal.pone.0058634. 23484044 PMC3590123

[15] Ho JG, Greco A, Rupnik M, Ng KK 2005 Crystal structure of receptor-binding C-terminal repeats from Clostridium difficile toxin A. Proc Natl Acad Sci U S A 102:18373–18378. doi:10.1073/pnas.0506391102. 16344467 PMC1317924

[16] Chumbler NM, Rutherford SA, Zhang Z, Farrow MA, Lisher JP, Farquhar E, Giedroc DP, Spiller BW, Melnyk RA, Lacy DB 2016 Crystal structure of Clostridium difficile toxin A. Nat Microbiol 1:15002. doi:10.1038/nmicrobiol.2015.2. PMC4976693 27571750

[17] Olling A, Hüls C, Goy S, Müller M, Krooss S, Rudolf I, Tatge H, Gerhard R 2014 The combined repetitive oligopeptides of Clostridium difficile toxin A counteract premature cleavage of the glucosyl-transferase domain by stabilizing protein conformation. Toxins (Basel) 6:2162–2176. doi:10.3390/toxins6072162. 25054784 PMC4113749

[18] Chen S, Wang H, Gu H, Sun C, Li S, Feng H, Wang J 2016 Identification of an essential region for translocation of Clostridium difficile toxin B. Toxins (Basel) 8:E241. doi:10.3390/toxins8080241. 27537911 PMC4999857

[19] Larabee JL, Krumholz A, Hunt JJ, Lanis JM, Ballard JD 2015 Exposure of neutralizing epitopes in the carboxyl-terminal domain of TcdB is altered by a proximal hypervariable region. J Biol Chem 290:6975–6985. doi:10.1074/jbc.M114.612184. 25614625 PMC4358121

[20] Tam J, Beilhartz GL, Auger A, Gupta P, Therien AG, Melnyk RA 2015 Small molecule inhibitors of Clostridium difficile toxin B-induced cellular damage. Chem Biol 22:175–185. doi:10.1016/j.chembiol.2014.12.010. 25619932

[21] Liu Z, Chen X 2016 Simple bioconjugate chemistry serves great clinical advances: albumin as a versatile platform for diagnosis and precision therapy. Chem Soc Rev 45:1432–1456. doi:10.1039/c5cs00158g. 26771036 PMC5227548

[22] Stoddart CA, Nault G, Galkina SA, Thibaudeau K, Bakis P, Bousquet-Gagnon N, Robitaille M, Bellomo M, Paradis V, Liscourt P, Lobach A, Rivard ME, Ptak RG, Mankowski MK, Bridon D, Quraishi O 2008 Albumin-conjugated C34 peptide HIV-1 fusion inhibitor: equipotent to C34 and T-20 in vitro with sustained activity in SCID-hu Thy/Liv mice. J Biol Chem 283:34045–34052. doi:10.1074/jbc.M805536200. 18809675 PMC2590714

[23] Abdeen SJ, Swett RJ, Feig AL 2010 Peptide inhibitors targeting Clostridium difficile toxins A and B. ACS Chem Biol 5:1097–1103. doi:10.1021/cb100209b. 20863124

[24] Mourez M, Kane RS, Mogridge J, Metallo S, Deschatelets P, Sellman BR, Whitesides GM, Collier RJ 2001 Designing a polyvalent inhibitor of anthrax toxin. Nat Biotechnol 19:958–961. doi:10.1038/nbt1001-958. 11581662

[25] Kudryashova E, Quintyn R, Seveau S, Lu W, Wysocki VH, Kudryashov DS 2014 Human defensins facilitate local unfolding of thermodynamically unstable regions of bacterial protein toxins. Immunity 41:709–721. doi:10.1016/j.immuni.2014.10.018. 25517613 PMC4269836

[26] Pruitt RN, Chumbler NM, Rutherford SA, Farrow MA, Friedman DB, Spiller B, Lacy DB 2012 Structural determinants of Clostridium difficile toxin A glucosyltransferase activity. J Biol Chem 287:8013–8020. doi:10.1074/jbc.M111.298414. 22267739 PMC3318759

[27] Zhang Y, Hamza T, Gao S, Feng H 2015 Masking autoprocessing of Clostridium difficile toxin A by the C-terminus combined repetitive oligo peptides. Biochem Biophys Res Commun 459:259–263. doi:10.1016/j.bbrc.2015.02.095. 25725153 PMC4426850

[28] Trevino SR, Schaefer S, Scholtz JM, Pace CN 2007 Increasing protein conformational stability by optimizing beta-turn sequence. J Mol Biol 373:211–218. doi:10.1016/j.jmb.2007.07.061. 17765922 PMC2084202

[29] Dempsey CE, Sessions RB, Lamble NV, Campbell SJ 2000 The asparagine-stabilized beta-turn of apamin: contribution to structural stability from dynamics simulation and amide hydrogen exchange analysis. Biochemistry 39:15944–15952. doi:10.1021/bi002044q. 11123921

[30] Eswar N, Ramakrishnan C 2000 Deterministic features of side-chain main-chain hydrogen bonds in globular protein structures. Protein Eng 13:227–238. doi:10.1093/protein/13.4.227. 10810153

