# Supplementary figures and images for: Intrinsic Toxin-Derived Peptides Destabilize and Inactivate Clostridium difficile TcdB
Source: mBio. 2017 May 16;8(3):e00503-17. doi: 10.1128/mBio.00503-17 (PMC5433098; doi:10.1128/mBio.00503-17)

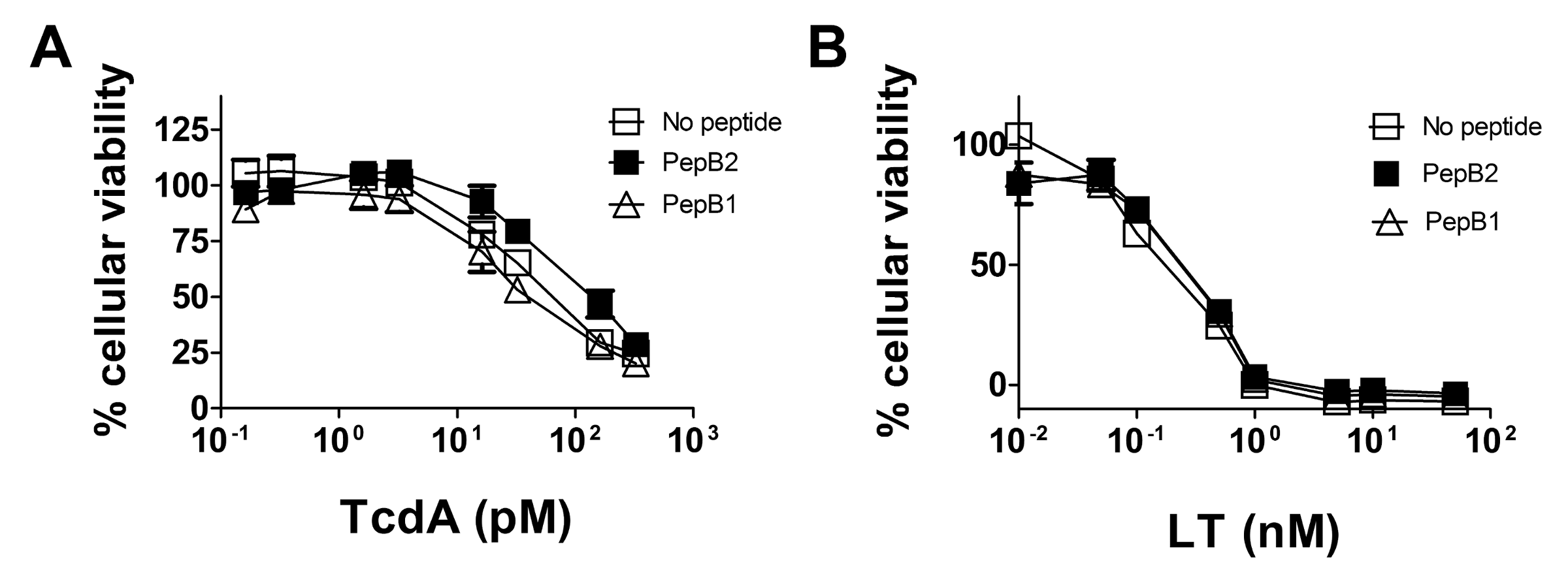

Supplement: FIG S1 [file mbo003173309sf1.tif]

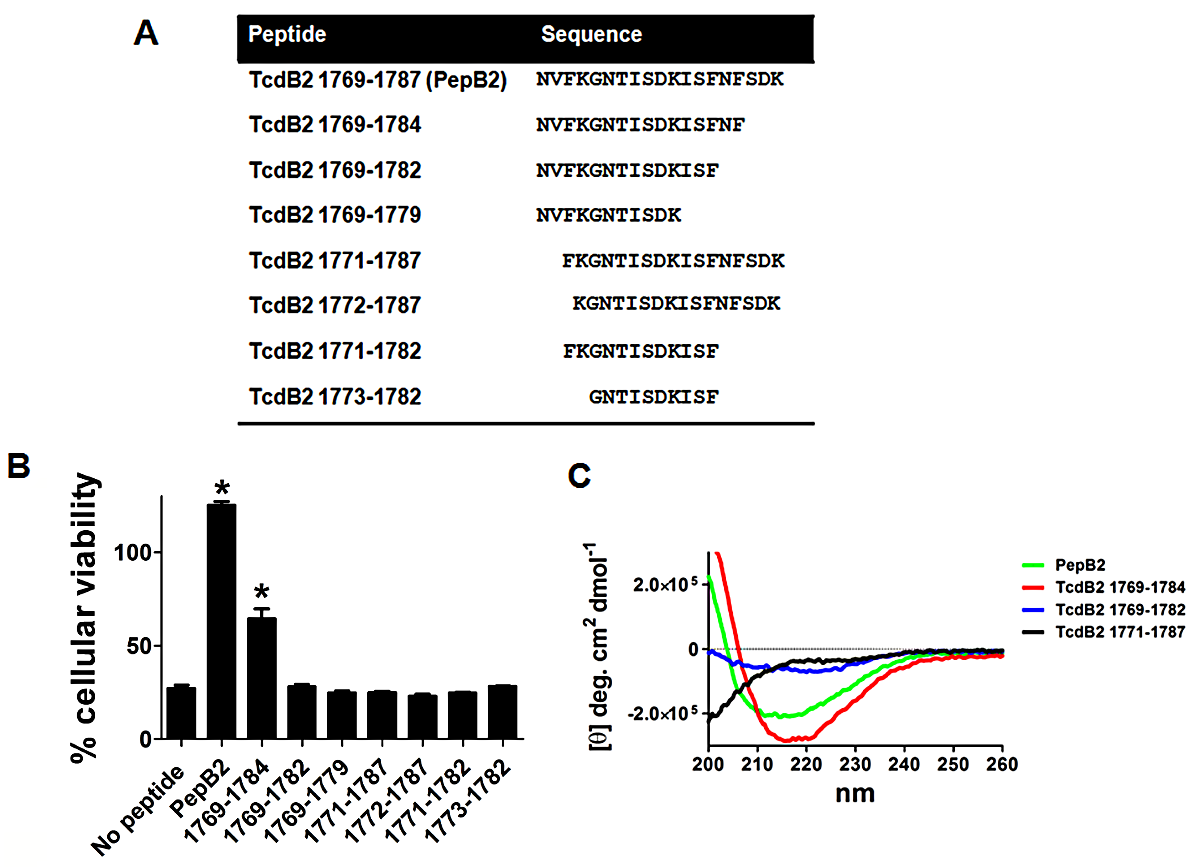

Supplement: FIG S2 [file mbo003173309sf2.tif]

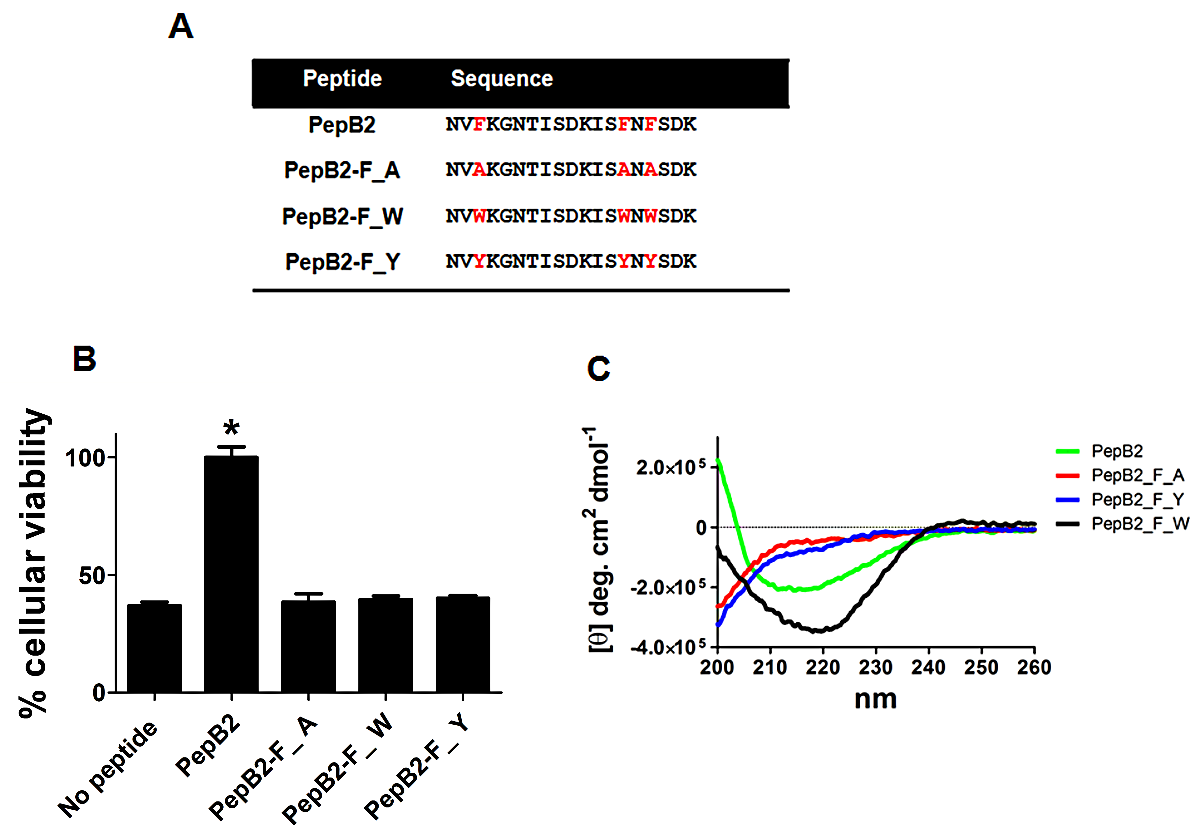

Supplement: FIG S3 [file mbo003173309sf3.tif]
